# Family Income, Parental Education and Chinese Preschoolers’ Cognitive School Readiness: Authoritative Parenting and Parental Involvement as Chain Mediators

**DOI:** 10.3389/fpsyg.2022.745093

**Published:** 2022-03-02

**Authors:** Xiaoying Xia

**Affiliations:** School of Early Childhood Education, Shanghai Normal University Tianhua College, Shanghai, China

**Keywords:** family income, parental education, parenting style, parental involvement, cognitive school readiness, mediation

## Abstract

This study examined the associations of family income and parental education with Chinese preschool children’s cognitive school readiness and the sequential mediating role of parenting style (i.e., authoritative parenting) and parental involvement in these relations. A total of 307 5–6 years old kindergarten children from Shanghai, China and their parents participated in the study. Using structural equation modeling method, the results indicated that parental education was directly related to children’s cognitive school readiness, while no direct relationship was found for family income. The link of parents’ education with children’s cognitive school readiness was sequentially mediated by authoritative parenting and home-based parental involvement. Authoritative parenting and parental involvement at home can be targeted by government administrators to effectively improve children’s cognitive school readiness skills. The theoretical and practical implications were discussed.

## Introduction

School readiness is perceived as a set of physical, cognitive, and social-emotional skills that children are expected to acquire to benefit from formal schooling (Pianta and Rimm-Kaufman, 2006). Cognitive aspects of school readiness (i.e., early literacy and numeracy skills, and language and cognitive development) are particularly powerful predictors of children’s academic performance in later school years (Duncan et al., 2007; Davis et al., 2016). Since the publication of the Coleman Report (Coleman, 1988), the influence of socioeconomic status (SES) on child outcomes has received extensive attention (Conger et al., 2010). Family SES is generally indexed by family income, parents’ educational attainment and occupational prestige. Research suggests that SES-based gap in children’s academic achievement emerges prior to kindergarten entry and the achievement gap is found to widen in subsequent school years (Brooks-Gunn and Markman, 2005; Duncan and Magnuson, 2005). In general, low-SES children perform less well than their higher-SES peers in school readiness outcomes (Ip et al., 2016). As different SES indicators are found to relate differently to children’s academic achievement (Chiu and Zeng, 2008), the unique effects of SES components on young children’s cognitive school readiness warrant further investigation.

A growing body of research has focused on the mechanisms underlying the link between family SES and children’s outcomes. For example, family income is found to indirectly relate to children’s cognitive competence through parents’ participation in cognitively stimulating activities (Yeung et al., 2002) and creating supportive and affectionate parenting environment (Mistry et al., 2008). However, most of previous researchers have studied the mediating role of parental involvement and parenting style separately. Parenting style and parental involvement, representing different aspects of the parenting process, are interwoven in daily parent–child interactions (Hindman and Morrison, 2012). Little work addresses the question as to whether parenting style is related to parental involvement and how the interrelationships between the two variables, in turn, affect children’s academic achievement.

In addition, individual SES indicators (i.e., family income and parents’ education) are found to be linked with children’s outcomes through different mechanisms (e.g., Bornstein and Bradley, 2003; Bukodi and Goldthorpe, 2013). For instance, researchers argue that family income might be more related to the material investment such as educational resources provided at home (Klein, 2011), while parental education might be more associated with psychological investment such as supportive parenting environment (Foster et al., 2005). In China, much of prior research has used SES as a composite and found positive relations with children’s early academic skills (e.g., Zhang et al., 2019). A recent study by Ren et al. (2020) found that parental education was related to Chinese preschoolers’ vocabulary, reading and math skills through both material investment (i.e., access to extracurricular activities) and parental involvement, while family income was associated with child outcomes only through extracurricular activities. More empirical evidence is needed to examine whether specific SES components (i.e., family income and parental education) relate to children’s school outcomes through different mechanisms.

### Family Income, Parental Education, and Children’s Cognitive School Readiness

Family income, parents’ education and occupation represent financial, human and social capital resources of the family separately, among which financial and human capital (i.e., family income and parents’ education) have been identified as primary factors for children’s cognitive outcomes (Bornstein and Bradley, 2003; Downer and Pianta, 2006). Previous studies using SES as a composite construct demonstrate positive associations between family SES and Chinese preschool children’s literacy, reading, math and cognitive skills (Ip et al., 2016; Li et al., 2016; Zhang et al., 2019). Nevertheless, findings on the relationship between individual SES indicators and children’s school outcomes are less conclusive. Some research suggests that children from households with more financial difficulties tend to show poorer cognitive and academic competence than those from families with higher-earned parents (Chatterji, 2006). However, other researchers found that family finance has weak effects on children’s academic achievement (Yeung and Conley, 2008). Besides, parents’ educational attainment is consistently found to be a stronger predictor of children’s cognitive outcomes than family income in numerous studies (e.g., Vasilyeva et al., 2018).

In addition, there is evidence that the strength of the relationship between family SES and children’s academic performance varies across different socioeconomic and cultural contexts. For example, a meta-analysis study reports a weak relationship between SES and children’s academic outcomes in developing countries and suggests that the strength of the relationship is stronger in economically developed countries (Kim et al., 2019). In the Chinese context, the income gap has largely widened since the reform and opening-up policy issued in 1978 and the educational level of Chinese parents has largely increased, especially in urban areas of China. Against this background, parents are capable of investing more resources to children’s education and dedicate more attention to their children’s development. Furthermore, Chinese parents attach much importance to children’s academic performance and tend to take their children’s academic success as a family honor (Huntsinger and Jose, 2009). These beliefs might motivate Chinese parents to participate in children’s education and create favorable learning environment regardless of their socioeconomic background (Wang and Sheikh-Khalil, 2014). Given that different elements of family SES convey different conceptual meanings (Conger and Donnellan, 2007), it is interesting to examine their unique pathway of influence on young children’s school outcomes in the Chinese context.

### Theoretical Framework for the Mediation Model

Much of the effects of family SES on children’s development outcomes can be attributed to the proximal family processes (Bradley and Corwyn, 2002; Conger and Donnellan, 2007). Parents’ cognitive stimulation and affective support are found to be positively related to children’s academic achievement (Bradley and Corwyn, 2004). Prior research with Western samples suggests that the role of family economic background is magnified and that positive parenting and parent–child daily activities have stronger influences on children’s development (Heckman and Mosso, 2014).

According to Family Investment Model (FIM), parents from higher-SES families possess more economic, social and human resources than those from lower-SES households, thus providing more investment in their children’s development (Duncan and Magnuson, 2003). In general, parents’ investment include a variety of forms, from providing cognitively stimulating materials such as toys and storybooks; involving children in cognitively enriching activities such as visiting museums, zoos, reading books, singing songs, and playing games; to creating an emotional and responsive parenting climate (Gershoff et al., 2007; Albarran and Reich, 2014; Sonnenschein and Sun, 2017). In contrast, economically disadvantaged parents may spend more money on the immediate needs of the family rather than on the development of their children (Bradley and Corwyn, 2002).

Another theoretical framework accounting for the links between family SES and children’s development is the Family Stress Model (FSM). This theory posits that low economic status may impose pressure on parents’ emotions, behaviors, and practices, which will lead to negative parenting behaviors such as low warmth and severe punishment of parents, which in turn hinders children’s cognitive development (Conger and Donnellan, 2007). For example, low-SES parents might suffer from emotional distress as a result of economic pressure and they are more likely to practice ineffective parenting with less warmth and responsiveness (Belsky et al., 2012; Vasilyeva et al., 2018), which in turn negatively impacts children’s cognitive development.

### Family Income, Parental Education, and Children’s Cognitive School Readiness Mediated Through Parenting Style

Parenting style is defined as a general child-rearing pattern comprising parental beliefs and attitudes toward their children (Steinberg et al., 1992). There are primarily three patterns of parenting styles: authoritarian, authoritative, and permissive parenting (Baumrind, 1967). Authoritative parenting characterized by warmth, responsiveness, and democracy, is generally linked with better academic outcomes (Merz et al., 2015). For example, authoritative parents are found to have preschool children with higher language and literacy competence (Roopnarine et al., 2006). In contrast, authoritarian parenting characterized by high control and strict demands but low warmth and responsiveness is negatively linked with children’s school readiness outcomes (Kessler, 2002). In China, recent work on parenting style has found similar results. For instance, Ren and Edwards (2017) reported authoritative parenting positively but authoritarian parenting negatively related to preschool children’s pre-academic skills.

There are pronounced disparities in parenting strategies between parents from different social classes (Lareau, 2003). Lower-SES parents tend to suffer more financial stress, which will probably lead to disrupted parenting, such as harsh and punitive parenting and low warmth and responsiveness (McLoyd, 1990). Empirical research by Hoff et al. (2002) found that mothers from low-SES families tend to be more controlling and restrictive than those from higher-SES households. Higher-SES parents are more likely to display authoritative parenting style characterized by warmth and responsiveness to their children (Hart et al., 2003). More recent research by Lombardi and Dearing (2021) found that family income was indirectly related to 5–6 years old children’s math school readiness skills through maternal support of children’s numeracy skill acquisition. These results indicate that parenting style is a potential mediator in the relationship between family SES and children’s cognitive school readiness. In addition, research suggests that highly educated parents are less influenced by psychological distress resulting from the economic disadvantage than those with lower educational level (Grzywacz et al., 2004). Thus, parents’ education is more related to parents’ parenting style than family income.

### Family Income, Parental Education, and Children’s Cognitive School Readiness Mediated Through Parental Involvement

Parental involvement refers to a variety of parenting behaviors to promote children’s learning and development, such as playing games with children, taking children to museums and libraries, and parent–child reading activities (Hill and Tyson, 2009; Wang and Sheikh-Khalil, 2014). Parental involvement is consistently found to be an important contributor to children’s cognitive and academic outcomes (Yeung et al., 2002; Gershoff et al., 2007). For example, empirical evidence indicates that parents’ participation in home-based literacy, numeracy and other cognitive enriching activities predict children’s cognitive development in the early years (Kiernan and Huerta, 2008; Sonnenschein and Sun, 2017). Also, research sampling Chinese preschoolers found that parents’ involvement at home positively predicted preschool children’s school readiness (Lau et al., 2011). Moreover, Deslandes et al. (1999) even report that parental involvement is a stronger predictor of children’s school performance than parenting style or family background.

A body of research has documented the link of family income and parental education with parental involvement (e.g., Wolf and McCoy, 2019). For example, research sampling Chinese children found that high-SES parents participate in more home-based activities than their lower-SES counterparts (Deng et al., 2017). Parents of low SES families may feel the pressure of life and are more likely to suffer negative emotions and family conflict, which in turn reduces their educational participation behavior (Camacho-Thompson et al., 2016). In addition, parental involvement has been identified as an important mediator in the associations between family SES and children’s early academic outcomes (Conger et al., 2010). For instance, family income and parents’ education were found to predict parents’ educational involvement, which in turn predicted preschool children’s early literacy, numeracy and cognitive skills (Wolf and McCoy, 2019). Specifically, in families of high SES, parents are more involved in their children’s learning activities, thus promoting the academic development of their children. In contrast, children from lower SES households are less exposed to cognitively stimulating materials and experiences, which in turn hinder their cognitive development (Gershoff et al., 2007). An earlier study by Smith et al. (1997) found that the mediation effect of home environment on the associations of family income and parents’ education with children’s academic achievement was stronger for parents’ education than for family income.

### The Chain Mediation of Parenting Style and Parental Involvement

Parenting style describes parents’ attitudes toward children, while parental involvement practices refers to specific behaviors that parents use to socialize their children (Darling and Steinberg, 1993). The two variables cannot be used interchangeably, as they represent different aspects of the family process for preschool children. Darling and Steinberg (1993) suggest that parenting style and parenting practice should be considered simultaneously in examining children’s development outcomes.

Prior research has suggested that parents with high authoritativeness are more likely to get involved in home-based education of their children. For example, Steinberg et al. (1992) found that parents who exhibit higher authoritative parenting tend to get more involved in their adolescent children’s education, which in turn is positively related to children’s academic achievement. In addition, other studies found support for the mediating role of parental involvement in the link between authoritative parenting and children’s school achievement. For instance, Bingham et al. (2017) found that authoritative parenting was indirectly related to young children’s language skills through the quality of home literacy practices. In their work, authoritative parents provided richer literacy practices at home, such as instruction of literacy skills and shared parent–child reading, whereas authoritarian parenting is a negative predictor of parents’ literacy involvement practices. These findings suggest that the positive effects of authoritative parenting may be mediated by more specific parenting behaviors (Patterson and Yoerger, 1991). Nevertheless, most of the existent research has focused either on parenting style or on parental involvement separately (Mistry et al., 2002; Sonnenshein and Munsterman, 2002; Aram and Levin, 2004; Davis-Kean et al., 2021). There is a need to consider authoritative parenting and parental involvement simultaneously in examining children’s school performance.

### The Present Study

**FIGURE 1:**
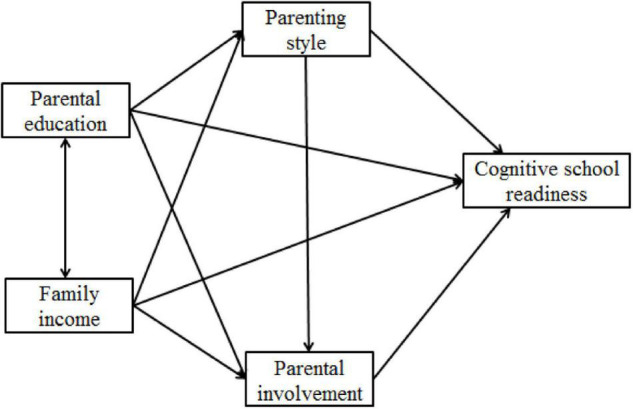
The hypothesized model of the relation between parental education and family income and children’s cognitive school readiness, mediated through parenting style and parental involvement.

As individual elements of family SES are reported to differentially affect children’s school outcomes, it is necessary to consider SES indicators as separate constructs in examining children’s development. Based on theoretical models of FIM and FSM, the effect of SES is interwoven with family process variables. Parenting style and parental involvement are two potential mediators in the link of SES with children’s academic outcomes. Recent researchers (Liu et al., 2020) suggest that family income tends to be more related with parents’ material investment (e.g., books and toys) and parental education tends to be more associated with psychological investment (e.g., parental involvement). Thus, this study aimed to test a sequential mediation model of how family income and parental education relate to children’s cognitive school readiness through parenting style and parental involvement. The hypothesized conceptual model for the study is presented in Figure 1. The specific research questions guiding this study are as follows: (1) What are the relationships among family income, parental education and Chinese preschoolers’ cognitive school readiness? It is hypothesized that children who have higher-earned and higher-educated parents show higher levels of cognitive school readiness. Parental education has a stronger relationship with children’s cognitive school readiness than family income. (2) Do parenting style and parental involvement sequentially mediate the relationship between family income and parental education and children’s cognitive school readiness? It is hypothesized that authoritative parenting and parental involvement sequentially mediate the relationship between family income and parental education and children’s cognitive school readiness.

## Materials and Methods

### Participants

The target population in this study was 5–6 years old senior kindergarteners in Shanghai, who will enter elementary school in the fall semester of the 2015–2016 school year. Chinese kindergartens offer services for 3–6 years old children, with 3–4 years old entering junior class, 4–5 years old entering middle class, and 5–6 years old entering senior class. Limited time and labor make it hard to conduct large-scale studies across kindergartens in all districts of Shanghai. Considering this, *Huangpu* district and *Zhabei* district were purposefully selected, as they represent different levels of economic strength in Shanghai. Principals of the potential kindergartens in the two districts were contacted through emails about the possibility of undertaking studies in their kindergartens. Eventually, four kindergartens located in the two administrative districts were involved in the study including two higher-SES and two lower-SES kindergartens.

In total, 324 of the targeted 341 kindergarten children and their parents participated in the study with a return rate of 95%. Listwise deletion was used to delete cases when the analyses involved variables that had missing values, yielding a final sample of 307 Chinese parents (mean age = 35.77, SD = 4.65) and their kindergarten children (mean age = 5.53, SD = 0.50). All the child participants are normal children without special needs reported by their parents, among which, 5 years olds accounted for 46.9% (*n* = 144) and 6 years olds accounted for 53.1% (*n* = 163). Boys and girls are almost equally distributed, accounting for 49.8 and 50.2%, respectively. Two thirds of the respondents were mothers (65.1%). It is noted that almost one third of the participants were fathers. Most of the responds obtained a college degree or above (26.7% finished 3-year college, 45.3% obtained bachelors’ degree, and 11.7% obtained masters’ degree and above). According to Shanghai Municipal Statistics Bureau (2015), the average per capita disposable income of urban households is *RMB* 47,710 a year (*RMB* 3,975 a month). Regarding family economic status, 31.3% families earned a monthly income below *RMB* 10,000 ($1,450); 53.3% families reported a monthly income ranging from *RMB* 10,001 to 30,000 ($4,350); 15.7% families had monthly income higher than *RMB* 30,001.

### Measures

#### Family Socioeconomic Status

Parents’ education was assessed based on a 6-point scale: 1 = junior middle school and below; 2 = high school/vocational school; 3 = associate degree; 4 = bachelor’s degree; and 5 = master’s degree or above. The average of maternal and paternal education scores was calculated to represent the variable of parental education. The monthly family income was evaluated on a 6-point scale: 1 = *RMB* 3,000 ($435) and below; 2 = *RMB* 3,001–6,000 ($870); 3 = *RMB* 6,001–10,000 ($1,450); 4 = *RMB* 10,001–30,000 ($4,350); 5 = *RMB* 30,001–50,000 ($7,250); 6 = *RMB* 50,001 and above. Higher scores represent higher income and parental education of the family. The two indicators of family SES were used separately in the present study.

#### Parenting Style

The Parenting Styles and Dimensions Questionnaire short version (PSDQ; Robinson et al., 2001) was employed to measure parents’ parenting style. The PSDQ has been widely used by researchers and demonstrates desirable reliability and validity (Robinson et al., 2001). The scale uses a 5-point Likert format (1 = never, 2 = once in a while, 3 = about half of the time, 4 = very often, 5 = always) to assess the frequency of parents’ child-rearing behaviors and attitudes. The original questionnaire consists of three dimensions: authoritative (15 items), authoritarian (12 items), and permissive (5 items). Previous research suggests that permissive parenting style is not applicable in the Chinese cultural context (Wu et al., 2002). In addition, the subscales of authoritarian parenting (i.e., physical coercion, verbal hostility, and non-reasoning and punitive) are found to have mixed results on Chinese preschool children’s outcomes (Li and Xie, 2017). They found that parents’ non-reasoning/punitive strategies negatively associated with Chinese preschoolers’ cognitive competence, while a positive relationship was identified for verbal hostility. Hence, only authoritative dimension was considered in this study. This dimension measures how often parents displayed warmth and acceptance, reasoning and induction, and democratic participation, including items such as “show sympathy when the child is hurt or frustrated,” “give child reasons why rules should be obeyed,” and “take child’s desire into account before asking him/her to do something.” One parent of the child completed the questionnaire by assessing how often they showed the parenting behaviors described in the scales. The mean of the 15 authoritative items was calculated for each parent participant, with a higher score indicating stronger tendency to authoritative parenting. In the present study, the Cronbach’s alpha was 0.87 for authoritative parenting.

#### Parental Involvement

The Family Involvement Questionnaire (FIQ) (Fantuzzo et al., 2000) was used to measure parents’ involvement practices. The FIQ was a multi-dimensional questionnaire and it was empirically designed for preschool, kindergarten, and first-grade children and their parents. In addition, the FIQ has been validated in numerous early childhood studies (Fantuzzo et al., 2004; Wanders et al., 2007), in which it was found to have strong predictive power for children’s school outcomes. In particular, the questionnaire has been translated and adapted to use among Chinese populations (Xia et al., 2021). This questionnaire is composed of 36 items based on a 4-point scale (1 = rarely, 2 = sometimes, 3 = often, 4 = always). There are three dimensions in the questionnaire: school-based involvement, home-based involvement, and home-school conferencing. As prior research suggests that Chinese parents get more involved in home-based activities than school-based activities and school-based activities have very limited influence on Chinese preschool children’s educational performance (Lau et al., 2011). Therefore, the present study focused exclusively on parents’ home-based involvement. Home-based involvement contains 11 items and measures how often parents are involved in educational activities at home to promote children’s learning and development, including items such as “I take my child to places in the community to learn special things” or “I spend time with my child working on reading/writing skills.” All the items in the home-based subscale were averaged to represent parents’ involvement at home. The Cronbach’s alpha value was 0.88 for the home-based involvement subscale.

#### Children’s School Readiness

The Early Development Instrument was used to measure children’s school readiness (EDI; Janus and Offord, 2007). The EDI is a 103-item instrument measuring children’s five development domains: physical health and well-being, social competence, emotional maturity, language and cognitive development, and communication skills and general knowledge. The Chinese version of the EDI has been validated in Chinese preschool children and presented desirable psychometrical soundness (Ip et al., 2013). Compared with other direct assessment of children’s school readiness, the EDI is easier to administer, especially for large populations (Janus and Offord, 2007). The EDI is completed by teachers based on their daily observations. In this study, the language and cognition subscale was used to assess children’s cognitive school readiness, which consists of 26 items measuring children’s basic and advanced literacy skills, numeracy skills, and interest in literacy, numeracy and memory. In this domain, all answers were scored on a 2-point scale: “yes” if a child possesses a skill, and “no” if he or she does not. All binary items were scored as 0 or 10 (Janus and Offord, 2007). The average score for the language and cognition subscale was calculated to represent children’s cognitive school readiness. Higher scores indicate better cognitive school readiness. In the present study, the Cronbach’s alpha was 0.87 for the language and cognition subscale.

### Data Collection

With approval from the IRB in the university and permission from the kindergartens, the investigator visited the principals of the four schools and explained the purpose and procedures of the study. Families were contacted by letters and asked whether they were willing to participate in the study. If parents agreed to participate in this study, they signed their names on the informed consent form. One parent of the child was asked to fill out the parenting questionnaire about their family background information, parenting style and parental involvement. The survey took approximately 15 min to complete. In addition, participants were assured that their responses and their children’s school readiness outcomes would be kept strictly confidential and only serve purposes of academic research. Subsequent to parent data collection, the collection of teacher data was initiated in each kindergarten. EDI school readiness assessment forms were given to the kindergarten teachers to rate the children based on their observation. To minimize measurement errors by different raters, all of the participant teachers were trained in the use of the instrument. Based on the training materials provided by Offord Centre for Child Studies and the suggestions of Hong Kong researchers who translated and validated EDI in China (Ip et al., 2013), the investigator conducted two training sessions on the guidelines of assessment criteria. In the session, the researcher gave each teacher participant a teachers’ guide on the use of the EDI and explained the assessment criteria for all the items. In particular, several confusing items were clarified when teachers rated children. A particular warning was informed in the training session to eliminate too many “I don’t know” responses in the assessment for each child. After training, the investigator distributed all the EDI assessment forms to teachers and asked them to be familiar with all the items and the teacher guide. Also, the researcher left a cell phone number and email address to them in case they had problems about the instrument. The teachers of the child participants’ class rated each child’s readiness skills based on their daily observations and interactions within one month subsequent to the assessment training.

### Data Analysis

First, the descriptive statistics and bivariate correlations of the main variables were calculated using SPSS 26.0. Second, to test the hypothesis that family income and parental education directly and indirectly related to children’s cognitive school readiness through parenting style and parental involvement, Amos 22.0 was used to examine the Structural Equation Model (SEM) incorporating family income and parents’ education as predictors, parenting style and parental involvement as sequential mediators, children’s cognitive school readiness as outcome variable, and children’s age and sex as control variables. Amos uses a maximum likelihood method for obtaining estimates of the parameters. To determine the adequacy of the model fit, the following goodness-of-fit indexes were used to test the model fit: non-significant Chi-square (χ^2^) value (*p* > 0.05), Chi-square ratios between 1 and 3, the comparative fit index (CFI) >0.90, the Tucker-Lewis Index (TLI) >0.90, a root mean square error of approximation (RMSEA) <0.05, and standardized root mean square residual (SRMR) <0.08 (Kline, 2005).

## Results

### Descriptive Analysis

The means, standard deviation, and correlations for the main variables of the study were illustrated in Table 1. Parents’ perceived authoritative parenting ranged from 2.00 to 5.00 with a mean of 3.98 (SD = 0.52) and home-based involvement ranged from 1.27 to 4.00 with a mean of 2.84 (SD = 0.57). Parents of 5-year olds get more involved at home than 6-year olds, *t* = 2.10, *p* < 0.05. No significant differences were found for authoritative parenting or home-based involvement across children’s gender. Children’s cognitive school readiness ranged from 0.43 to 10 with a mean of 8.35 (SD = 1.66). Girls showed higher levels of cognitive school readiness than boys, *t* = −2.45, *p* < 0.05. There is no significant difference between 5 and 6 year old children in cognitive school readiness. Family income (*r* = 0.17, *p* < 0.01) and parental education (*r* = 0.18, *p* < 0.01) were positively associated with children’s cognitive school readiness. Family income was positively related to authoritative parenting (*r* = 0.18, *p* < 0.01) and home-based involvement (*r* = 0.22, *p* < 0.001). Parental education was positively related to authoritative parenting (*r* = 0.29, *p* < 0.001) and home-based involvement (*r* = 0.32, *p* < 0.001). These results suggest that parental education was more related to the parenting process variables than family income. Both authoritative parenting (*r* = 0.23, *p* < 0.001) and home-based involvement (*r* = 0.26, *p* < 0.001) were positively linked with children’s cognitive school readiness. These results suggest that authoritative parenting and parental involvement are potential mediators in the SES-cognitive school readiness relationship. It is noted that authoritative parenting is positively related to home-based involvement (*r* = 0.57, *p* < 0.001).

**TABLE 1:** Zero-order correlations of main variables.

Variable	2	3	4	5	6	7
1. Child age	−0.07	−0.02	–0.06	–0.07	−0.12*	0.10
2. Child gender	–	0.04	–0.01	–0.03	0.07	0.14*
3. Family income		–	0.63***	0.18**	0.22***	0.17**
4. Parental education			–	0.29***	0.32***	0.18**
5. Authoritative parenting				–	0.57***	0.23***
6. Home-based involvement					–	0.26**
7. Cognitive school readiness						–
Mean	–	3.77	3.46	3.98	2.84	8.35
SD	–	0.93	1.04	0.52	0.57	1.66

n = 307, *p < 0.05, **p < 0.01, ***p < 0.001.

### The Direct Relationship Between Family Income, Parental Education, and Children’s Cognitive School Readiness

**FIGURE 2:**
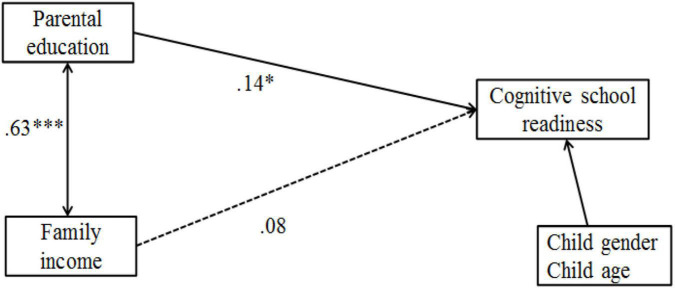
The direct model. *n* = 307. Dotted line was used for insignificant paths. ^∗^*p* < 0.05, ^∗∗∗^*p* < 0.001.

To examine the direct link of family income and parental education with children’s cognitive school readiness, a simplified SEM model involving family income and parents’ education as predictors and cognitive school readiness as the outcome variable was tested (see Figure 2). Child age and gender were control variables. Results suggested that the model fit the data well: Chi-square (χ^2^) value = 0.87, *p* > 0.05, CFI = 1.00, GFI = 0.99, TLI = 1.00, RMSEA = 0.00 (90% CI = 0.00–0.07). After controlling for children’s age and gender, only parents’ education directly and positively related to children’s cognitive school readiness (β = 0.14, *p* < 0.05) and family income was not directly associated with children’s cognitive school readiness (β = 0.08, *p* = 0.27).

### Parenting Style and Parental Involvement as Sequential Mediators

**FIGURE 3:**
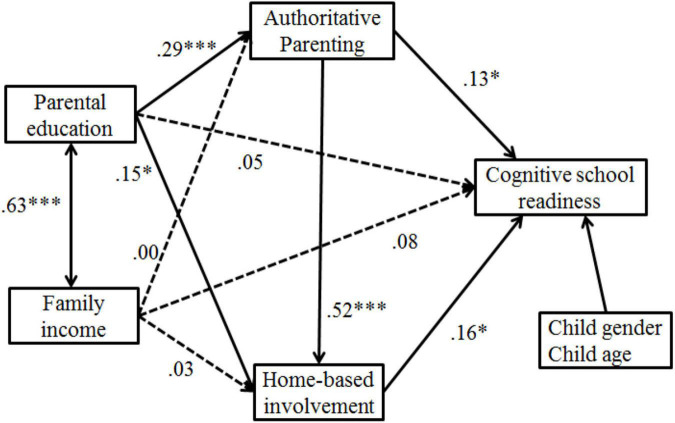
The sequential mediation model. *n* = 307. Dotted lines were used for insignificant paths. ^∗^*p* < 0.05, ^∗∗∗^*p* < 0.001.

A second SEM model was used to test the indirect path from parental education and family income to children’s cognitive school readiness through authoritative parenting and parental involvement. As shown in Figure 3, the sequential mediation model fit the data well, Chi-square (χ^2^) value = 11.42, *p* > 0.05, CFI = 0.99, GFI = 0.99, TLI = 0.98, RMSEA = 0.03 (90% CI = 0.00–0.075), SRMR = 0.038.

An investigation of the indirect pathway from parental education to children’s cognitive school readiness revealed three significant indirect paths. After controlling for child age and child gender, the path coefficients from parental education to authoritative parenting (β = 0.29, *p* < 0.001), from parental education to home-based involvement (β = 0.15, *p* < 0.05), from authoritative parenting to home-based involvement (β = 0.52, *p* < 0.001), from authoritative parenting (β = 0.13, *p* < 0.05) and home-based involvement (β = 0.16, *p* < 0.05) to children’s cognitive school readiness were all significant. The path coefficients from family income to authoritative parenting (β = 0.00, *p* = 0.97), from family income to home-based involvement (β = 0.03, *p* = 0.64), from family income to children’s cognitive school readiness (β = 0.08, *p* = 0.26) were not statistically significant after controlling for child age and child gender. It is noted that the direct link of parental education with cognitive school readiness was not significant after including authoritative parenting and parental involvement as sequential mediators (β = 0.05, *p* = 0.46). This result indicated that the effect of parents’ education on children’s cognitive school readiness was fully mediated by the sequential indirect effects of authoritative parenting and parental involvement.

The indirect path from parental education to children’s cognitive school readiness *via* authoritative parenting was statistically significant (standardized indirect effect = 0.038, *p* < 0.05). The indirect path from parental education to children’s cognitive school readiness *via* home-based involvement was statistically significant (standardized indirect effect = 0.024, *p* < 0.05). The indirect effect of parental education on children’s cognitive readiness *via* parenting style and parental involvement was statistically significant (standardized indirect effect = 0.024, *p* < 0.05). The total indirect effect of parental education on children’s cognitive school readiness was β = 0.086, *p* < 0.05. The indirect effect of authoritative parenting and home-based involvement accounted for 62.31% of the total effect of parental education on children’s cognitive school readiness.

## Discussion

The present study explored how individual family SES indicators directly and indirectly related to children’s cognitive school readiness skills. Parental education was directly related to children’s cognitive school readiness, yet no significant direct relation was found for family income. Authoritative parenting and parental involvement sequentially mediate the relationship between parental education and children’s cognitive school readiness. The hypothesis that parental education was indirectly related to children’s cognitive school readiness through parenting style and parental involvement was supported. However, it is unexpected that the direct and indirect links between family income and children’s cognitive readiness were not identified.

### Family Income, Parental Education, and Children’s Cognitive School Readiness

This study revealed that parental education was positively associated with children’s cognitive readiness. This finding is in line with the existing research, which demonstrated that children of highly educated parents perform better in cognitive competence than those with less-educated parents (Petterson and Albers, 2001). One explanation may be attributed to the different quality of parent–child interactions between low-educated and highly educated parents. In the Chinese cultural setting, higher-educated parents have benefited from their own higher levels of educational attainment and therefore put more emphasis on their children’s education and provide more support for children. On the other hand, parents with higher educational attainment are more likely to acquire and update positive child-rearing knowledge and thereby tend to employ positive parenting practices (Bornstein et al., 2003).

The present study found that parental education had a stronger relationship with children’s cognitive school readiness than family income. This finding concurs with a number of previous studies, which found that parental education is a stronger predictor of children’s academic achievement than family income (e.g., Raviv et al., 2004; Davis-Kean, 2005; Erola et al., 2016; Gonzalez et al., 2017; Luo and Zhang, 2017; Davis-Kean et al., 2021). For instance, parents’ education level accounted more variance of children’s early academic skills than family income (Vasilyeva et al., 2018). Higher-educated parents might be more knowledgeable about what children are learning at school and thus provide appropriate cognitively stimulating activities and emotionally supportive environment at home. In addition, it might be because parents’ education is more related to the quality of parent–child interactions and parents’ involvement in cognitively stimulating activities than family income. This might explain the stronger relationship between parents’ education and children’s cognitive school readiness than family income.

The insignificant link of family income with Chinese children’s cognitive school readiness concurs with a cross-cultural study on children’s math achievement, which indicates that family income is less linked to children’s math achievement in China than their peers in the United States (Tsui, 2005). In addition, a prior research on British children reported a weak direct effect of family financial resources on children’s development outcomes and the effect disappeared after family process variables (i.e., cognitively stimulating initiatives and parenting practices) were controlled for (Violato et al., 2011). In the present study, family income was not related to authoritative parenting and parental involvement. This confirms prior researchers’ argument that Chinese parents try to establish favorable learning environment for children regardless of their economic family background (Wang and Sheikh-Khalil, 2014). It seems that family income is less associated with parents’ psychological investment (i.e., parenting style and parental involvement) and has a trivial direct effect on preschool children’s cognitive school readiness in the Chinese context. On the other hand, this study was conducted in an economically advantaged city of China (i.e., Shanghai). The rich educational resources in the community are available to children, which can offset the scarce resources for children living in poverty.

### Parental Involvement and Parenting Style as Sequential Mediators

After accounting for the role of authoritative parenting and parental involvement, the direct path from parental education to children’s cognitive school readiness was not significant. This suggests that authoritative parenting and parental involvement fully mediated the association between parental education and cognitive school readiness. This result echoes previous empirical research by Wolf and McCoy (2019), which indicates that higher-educated parents are more likely to provide appropriate cognitive stimulation and create warm and supportive atmosphere at home, both of which contributes to children’s cognitive school readiness skills. Another research using SES as a composite also found the mediating effect of language and literacy stimulation by parents and supportive parenting atmosphere on the associations between family SES and children’s cognitive development outcomes (Mistry et al., 2008). FIM posits that higher-SES parents tend to invest more emotional and social capital in their children. Higher-educated parents are more likely to provide quality educational activities than those with less education (Ip et al., 2016). In contrast, low-SES parents with less education might be less confident in participating in children’s education (Tazouti and Jarlegan, 2016). On the other hand, the sequential mediation result also shows that authoritative parenting motivates home-based involvement, which subsequently promotes children’s cognitive school readiness. This finding echoes a recent study with sixth graders in Iran (Amani et al., 2020). In their study, authoritative parenting was found to positively relate to children’s academic achievement through parental education involvement. This result provides evidence that authoritative parenting contribute to children’s cognitive school readiness in part through higher levels of parental involvement.

Unexpectedly, no mediating effects of either authoritative parenting or home-based parental involvement were found for the relation between family income and children’s cognitive school readiness. These findings suggest that economic disadvantages do not necessarily constrain children’s acquisition of cognitive school readiness. It is also possible that family income is more linked with “material investment” rather than “psychological investment” (Liu et al., 2020, p. 230). Family income, representing a family’s financial conditions, is more related to the material resources provided at home, while parents’ educational attainment primarily captures the cultural capital of the family and is more associated with parents’ educational involvement and supportive learning atmosphere at home (Foster et al., 2005). A recent study by Ren et al. (2020) reported that family income was only indirectly associated with children’s academic school readiness through enrolling children in extracurricular activities rather than through participating in educational activities at home, but parents’ educational level was positively linked with their engagement in education. Another empirical study sampling Chilean preschool children found that family income was indirectly linked with children’s vocabulary performance through the living conditions of the family while parental education indirectly related to preschoolers’ vocabulary through both standard of living and cognitive stimulation practices (Coddington et al., 2014). These results provide evidence that family income and parental education influence children’s cognitive school readiness through different mechanisms. In this study, parents’ education has an impact on children’s cognitive school readiness through psychological investment (i.e., authoritative parenting and parental educational involvement), but these mediation paths for family income were not significant. Whether family income primarily influence children’s outcomes through finance-related material investment is worthy of further empirical research work. It is noteworthy that family income might be differently related to children academic and social outcomes. For example, a recent study by Ren et al. (2020) found that family income had a stronger relationship with children’s social skills while parental education had larger associations with children’s academic readiness outcomes. The direct and indirect link of individual SES indicators with diverse child outcomes can be replicated in further studies.

## Implications

This study is one of the few to examine the associations among individual SES components and children’s cognitive school readiness as well as the sequential mediating role of parenting style and parental involvement. The results confirmed the significance of parental education, authoritative parenting and parental involvement for children’s cognitive school readiness. The present study showed that parental education was significantly related to re children’s cognitive school readiness, while the direct link was not significant for family income. This finding suggests that early interventions need to be more focused on families with less-educated parents especially in urban areas of China, where economic wealth is relatively advanced. Also, the insignificant link of family income indicates that low-income does not necessarily constrain children’s cognitive school readiness development.

Positive authoritative parenting and parental involvement in family education played a full mediating role in the influence of parental education on children’s cognitive school readiness. The full mediation model implies the significance of family intervention programs targeted at enhancing parental involvement and positive parenting style, which can compensate for the scarce resources available to children in financially constrained families. Although disadvantaged in financial capital and cultural capital, low-SES parents can increase social capital by creating authoritative parenting atmosphere and actively participating in children’s education to offset the negative effects of socioeconomic advantages. Prior research suggests that intervention programs incorporating parents’ education and cognitive stimulation practices at home exerts stronger effect on children’s cognitive competence than money assistance (Nores and Barnett, 2010). Parents’ active educational involvement and emotional parenting atmosphere can reduce the negative effect of financial disadvantages. Preschools can establish parent training programs to help parents, especially lower-SES parents, create emotionally supportive environment and practice developmentally appropriate learning involvement. The schools can make full use of educational resources to popularize relevant knowledge to parents in the form of special lectures and theme activities, so that parents can realized the importance of investing time in children’s early development and learn to use authoritative parenting to promote the children’s cognitive school readiness. The administrative departments should intensify efforts to publicize the positive effects of authoritative parenting and quality educational involvement in the early childhood. It is necessary to carry out parental training programs regularly to help parents establish favorable parenting values and practices, especially those with less education to play a positive family function. Parents’ education is more strongly related to children’s cognitive school readiness than family income. This has important implications for the national welfare policy providing chances for those parents who want to obtain additional education. Better-educated parents can help children perform better in early cognitive school readiness.

## Limitations and Conclusion

There are several limitations of this study. First, the participants were only recruited in an economically advantaged city of China and therefore the sample cannot represent all Chinese families. Caution should be made when generalizing the results among all Chinese population. Stratified random sampling method can be used to elicit data from diverse regions across China to obtain a more representative sample. Second, all the parenting style and parental involvement were reported by parents. Direct observations may get a more objective assessment. Third, we did not include authoritarian parenting in the mediation model as mixed effects were found for the sub-dimensions of authoritarian parenting in the Chinese context. Further research can explore the mediating role of sub-dimensions in China or in other cultural settings. Fourth, due to the limited sample, this study did not distinguish mothers and fathers in analyzing the mediation relationship between family SES indicators and children’s cognitive school readiness. Whether maternal and paternal parenting style and parental involvement function differently in the SES-based gap in academic outcomes needs further investigation. Fifth, cross-sectional data was used in this study to test the process model. Longitudinal research design may disclose a more dynamic process of how family SES indicators relate to children’s cognitive school readiness.

Despite the limitations mentioned above, this study contributes to the existent literature on the relationship among SES indicators and young children’s cognitive school readiness and the sequential mediating role of both parenting style and parental involvement. Results of this study point to the importance of examining individual SES indicators and incorporating both parenting style and parental involvement in children’s development outcomes. It is found that positive parenting style and quality parental involvement in early childhood education can offset the negative effects of low economic disadvantages on children’ cognitive readiness skills. These results provide important implications for reducing the negative effects of social-economic disparities on children’s early cognitive school readiness.

## Data Availability Statement

The raw data supporting the conclusions of this article will be made available by the authors, without undue reservation.

## Ethics Statement

The studies involving human participants were reviewed and approved by University of the Pacific. Written informed consent to participate in this study was provided by the participants’ legal guardian/next of kin.

## Author Contributions

XX collected and analyzed the data, and wrote the manuscript independently.

## Conflict of Interest

The author declares that the research was conducted in the absence of any commercial or financial relationships that could be construed as a potential conflict of interest.

## Publisher’s Note

All claims expressed in this article are solely those of the authors and do not necessarily represent those of their affiliated organizations, or those of the publisher, the editors and the reviewers. Any product that may be evaluated in this article, or claim that may be made by its manufacturer, is not guaranteed or endorsed by the publisher.
